# Aneuploidy underlies brefeldin A-induced antifungal drug resistance in *Cryptococcus neoformans*

**DOI:** 10.3389/fcimb.2024.1397724

**Published:** 2024-06-20

**Authors:** Zhi-hui Zhang, Liu-liu Sun, Bu-qing Fu, Jie Deng, Cheng-lin Jia, Ming-xing Miao, Feng Yang, Yong-bing Cao, Tian-hua Yan

**Affiliations:** ^1^ Institute of Vascular Disease, Shanghai TCM-Integrated Hospital, Shanghai University of Traditional Chinese Medicine, Shanghai, China; ^2^ Department of Pharmacy, Shanghai Tenth People’s Hospital, Tongji University School of Medicine, Shanghai, China; ^3^ Laboratory Department, Jiangsu Province Hospital of Chinese Medicine, Nanjing, China; ^4^ Department of Physiology and Pharmacology, School of Basic Medicine and Clinical Pharmacy, China Pharmaceutical University, Nanjing, China

**Keywords:** *Cryptococcus neoformans*, aneuploidy, brefeldin A, antifungal drugs, *AFR1*, cross-resistance

## Abstract

*Cryptococcus neoformans* is at the top of the list of “most wanted” human pathogens. Only three classes of antifungal drugs are available for the treatment of cryptococcosis. Studies on antifungal resistance mechanisms are limited to the investigation of how a particular antifungal drug induces resistance to a particular drug, and the impact of stresses other than antifungals on the development of antifungal resistance and even cross-resistance is largely unexplored. The endoplasmic reticulum (ER) is a ubiquitous subcellular organelle of eukaryotic cells. Brefeldin A (BFA) is a widely used chemical inducer of ER stress. Here, we found that both weak and strong selection by BFA caused aneuploidy formation in *C. neoformans*, mainly disomy of chromosome 1, chromosome 3, and chromosome 7. Disomy of chromosome 1 conferred cross-resistance to two classes of antifungal drugs: fluconazole and 5-flucytosine, as well as hypersensitivity to amphotericin B. However, drug resistance was unstable, due to the intrinsic instability of aneuploidy. We found overexpression of *AFR1* on Chr1 and *GEA2* on Chr3 phenocopied BFA resistance conferred by chromosome disomy. Overexpression of *AFR1* also caused resistance to fluconazole and hypersensitivity to amphotericin B. Furthermore, a strain with a deletion of *AFR1* failed to form chromosome 1 disomy upon BFA treatment. Transcriptome analysis indicated that chromosome 1 disomy simultaneously upregulated *AFR1*, *ERG11*, and other efflux and *ERG* genes. Thus, we posit that BFA has the potential to drive the rapid development of drug resistance and even cross-resistance in *C. neoformans*, with genome plasticity as the accomplice.

## Introduction

In 2022, the World Health Organization released the first-ever “most wanted” list of health-threatening fungi, and *Cryptococcus neoformans* is at the top of the list (WHO, 2022). *C. neoformans* lives in the environment throughout the world. The fungus is typically found in soil, on decaying wood, in tree hollows, or in bird droppings. Inhalation is the principal route of entry into human body. After inhalation, *C. neoformans* may disseminate to the brain and meninge, resulting in cryptococcal meningitis, which has high mortality (Zhao et al., 2023). Yet, infections are rare in healthy people. In most cases, cryptococcosis caused by *C. neoformans* occurs in people with compromised immune systems, particularly people with advanced HIV/AIDS. Annually, approximately 152,000 cases of cryptococcal meningitis occur among people with HIV/AIDS worldwide, resulting in nearly 112,000 deaths (Rajasingham et al., 2017).

Fungi are eukaryotes and share many of the same basic cell structures and machinery with mammalian cells. Therefore, a potential antifungal agent may cause serious side effects due to off-target effects. Currently, only four classes of antifungal drugs are available: polyenes [e.g., amphotericin B (AMB)], azoles [e.g., fluconazole (FLC)], 5-flucytosine (5FC), and echinocandins (e.g., caspofungin). AMB is fungicidal. It acts by binding to membrane sterols, primarily ergosterol, which leads to the formation of pores that allow leakage of cellular components (Ghannoum and Rice, 1999). FLC is fungistatic. It prevents the conversion of lanosterol to ergosterol by inhibiting the lanosterol 14-α-demethylase encoded by *ERG11* involved in the biosynthesis of ergosterol. Ergosterol is fungal-specific and is the primary sterol in the fungal cell membrane. The depletion of ergosterol and the accumulation of precursors affect the fluidity of the lipid bilayer and slow fungal growth (Ghannoum and Rice, 1999). 5FC is a prodrug. 5FC is taken up by fungal cells by cytosine permease. Then, 5FC is deaminated to 5-fluorouracil (5FU) by cytosine deaminase. Subsequent metabolites of 5FU inhibit DNA and RNA synthesis (Ghannoum and Rice, 1999). Echinocandins are non-competitive inhibitors of β-1,3-glucan synthase (Douglas et al., 1997). Inhibition of the major fungal cell wall component β-(1,3)-glucan biosynthesis leads to growth inhibition or death owing to imbalance in osmotic pressure (Letscher-Bru and Herbrecht, 2003). However, *Cryptococcus* spp. are intrinsically resistant to echinocandins (Denning, 2003), although cryptococcal β-1,3-glucan synthase is essential and is potently inhibited by echinocandins in cell-free assays (Thompson et al., 1999; Maligie and Selitrennikoff, 2005).

Current understanding of the antifungal resistance mechanisms is very limited. Resistance to AMB remains extremely rare. This might be due to the severe fitness cost accompanied by gain of AMB resistance (Vincent et al., 2013). There is still no clear molecular mechanism of AMB resistance. Potential mechanisms include altered sterol composition of membranes, the regulation of oxidative stress, and alterations of the fungal cell wall (Carolus et al., 2020). Loss-of-function mutations of genes involved in the cellular uptake or intracellular metabolism of 5FC contribute directly to 5FC resistance (Hope et al., 2004; Chang et al., 2021). Recent studies indicate that mutations of some genes, including *UXS1*, contribute indirectly to 5FC resistance (Billmyre et al., 2020; Chang et al., 2021). In addition to genetic mutation, a recent study indicates that aneuploidy also causes 5FC resistance. Exposure of *C. neoformans* lab strain H99 to 5FC selects Chr1 disomy (Chr1x2) mutants. Chr1x2 confers 5FC resistance via the increased copy number of *AFR1*, which encodes efflux pump. However, it is still unknown how *AFR1* is involved in 5FC resistance (Chang et al., 2021). By virtue of good safety, oral bioavailability, and relatively low cost, FLC is the most widely used antifungal drug. However, resistance of *C. neoformans* clinical isolates to FLC is progressively increasing. The rates of resistance to FLC were 7.3% for the years 1997 to 2000, 10.9% for the years 2001 to 2004, and 11.7% for the years 2005 to 2007 (Pfaller et al., 2009). Resistance to FLC is generally due to increased efflux or alteration of genes involved in the ergosterol biosynthesis pathway (Fisher et al., 2022). Although point mutations of *ERG11* cause FLC resistance, in most cases, increased copy number and thereby expression of *ERG11* mediated by formation of Chr1x2 is the major mechanism of FLC resistance developed both *in vitro* and *in vivo* (Sionov et al., 2010; Zafar et al., 2019; Yang et al., 2021a). In addition to *ERG11*, *AFR1* is also on Chr1, and amplification of *AFR1* also contributes to Chr1x2-mediated FLC resistance (Sionov et al., 2010). In the diploid fungal pathogen *Candida albicans*, *ERG11* and *TAC1* are on the left arm of Chr5. *TAC1* encodes a transcription factor that positively regulates the efflux pump genes *CDR1* and *CDR2*. More than 50% of clinical FLC-resistant isolates of *C. albicans* bear amplification of the left arm of Chr5 (Selmecki et al., 2006; Selmecki et al., 2008).Therefore, in both *C. neoformans* and *C. albicans*, exposure to FLC selects particular aneuploidy (Chr1x2), which simultaneously upregulates *ERG11* and directly or indirectly upregulates efflux pump genes, resulting in FLC resistance. In *C. neoformans*, exposure to 5FC also selects Chr1x2.

In addition to antifungal-induced aneuploidy formation, some *C. neoformans* isolates obtained directly from the cerebrospinal fluid of HIV/AIDS patients, as well as some isolates obtained after passage in mice, were also aneuploid or displayed altered electrophoretic karyotypes (Fries et al., 1996; Hu et al., 2011), implying that aneuploidy formation of *C. neoformans* also happens *in vivo*; however, the driving force of genome instability *in vivo* is still unknown.

The endoplasmic reticulum (ER) is a ubiquitous organelle of eukaryotes. It is a major protein folding compartment for secreted, plasma membrane, and organelle proteins. When the capacity of the ER to fold proteins becomes saturated, ER stress occurs. ER stress usually activates a signaling network named the unfolded protein response (UPR), which is an adaptive reaction that eliminates ER stress. Chronic or irreversible ER stress triggers apoptosis (Luoma, 2013).

Tunicamycin (TUN) and brefeldin A (BFA) are commonly used chemical inducers of ER stress. TUN acts by blocking the formation of N-glycoside linkages to proteins, causing the accumulation of unfolded proteins in the cell ER (Wu et al., 2018). BFA acts by blocking the transport of proteins from ER to the Golgi apparatus, leading to the accumulation of secretory proteins within the ER (Citterio et al., 2008). BFA is an uncompetitive inhibitor of the Sec7 domain-catalyzed nucleotide exchange on Arf (ADP-ribosylation factor) proteins, which are major regulators of membrane traffic in the cell. The GDP/GTP exchange of Arf proteins is stimulated by guanine nucleotide-exchange factors (GEFs). GEFs carry a catalytic Sec7 domain. BFA binds to the Arf GDP–Arf GEF complex (Robineau et al., 2000). In *Saccharomyces cerevisiae*, *GEA1*, *GEA2*, and *SEC7* encode ARF exchange factors: Gea1p, Gea2p, and Sec7p, respectively. Overexpression of *SEC7* and *GEA2* confers resistance to BFA-inhibited secretion (Peyroche et al., 1999). The ortholog of *S. cerevisiae GEA1* and *GEA2* in *C. neoformans* is *GEA2/CNAG_07514*, which is on Chr3. The *C. neoformans SEC7/CNAG_01126* is on Chr5. In addition, BFA is also a substrate of efflux pumps (Diwischek et al., 2009).Among the three classes of antifungal drugs available for the treatment of cryptococcosis, both FLC and 5FC select Chr1 disomy, which causes cross-resistance to FLC and 5FC (Sionov et al., 2010; Chang et al., 2021; Yang et al., 2021a). However, it is unknown whether other stresses, particularly ER stress, can cause antifungal resistance in *C. neoformans*. In *S. cerevisiae* and *C. albicans*, exposure to TUN results in mainly ChrRIIx2 and Chr2x3, respectively. *S. cerevisiae* ChrRII genes *ALG7*, *PRE7*, and *YBR085C-A*, and *C. albicans* Chr2 genes *ALG7, RTA2*, and *RTA3* are associated with TUN resistance (Beaupere et al., 2018; Yang et al., 2021b).

In this study, we investigated the impact of BFA on genome plasticity and consequently alterations of antifungal resistance in *C. neoformans*. We found that even exposure to the sub-inhibitory concentration of BFA was sufficient to select aneuploid adaptors with diverse karyotypes. Exposure to a high amount of BFA also selected aneuploids. The same karyotypes, Chr1 disomy and Chr3 disomy, were recurrently isolated under both weak and strong selective conditions. Chr1 disomy, but not Chr3 disomy, conferred cross-resistance to FLC and 5FC. However, drug resistance was unstable, due to the intrinsic genome instability in aneuploids. Mechanistically, two different genes, *AFR1* on Chr1 and *GEA2* on Chr3, were associated with BFA resistance. *AFR1* was also associated with FLC resistance and hypersensitivity to AMB. At the chromosome level, Chr1 disomy upregulated multiple genes associated with FLC resistance and 5FC resistance, including efflux pump genes and *ERG* genes. A strain with a deletion of *AFR1* failed to form Chr1 disomy upon BFA treatment. Thus, we posit that genome plasticity of *C. neoformans* enables rapid adaptation to BFA and is the underlying mechanism of serendipitous development of cross-resistance to two of the three major anti-cryptococcal drugs.

## Materials and methods

### Strains and growth conditions

*C. neoformans* lab strain H99 was used as the wild-type strain. Stock culture was preserved in 25% glycerol and maintained at −80°C. Cells were routinely grown in Yeast extract–Peptone–Dextrose (YPD) media [1% (w/v) yeast extract, 2% (w/v) peptone, and 2% (w/v) D-glucose] at 30°C in a shaking incubator at 150–200 rpm. For solid medium, 2% (w/v) agar was added. Drugs were dissolved in dimethyl sulfoxide (DMSO) and stored at −20°C.

### Growth curves

Strains were streaked from a −80°C freezer to YPD agar and incubated at 30°C for 72 h. Cells were suspended in YPD broth. Cell densities were adjusted to 2.5 × 10^3^ cells/mL in YPD broth with or without BFA in a 96-well plate. The plate was incubated at 30°C. OD_595_ was monitored in a Tecan plate reader (Infinite F200 PRO, Tecan, Switzerland) at 15-min time intervals for 72 h. Data are presented as the mean ± SD of three biological replicates.

### Spot assay

Cells were suspended in distilled water and adjusted to 1 × 10^7^ cells/mL. Three microliters of 10-fold serial dilutions was spotted on YPD with or without drugs. The plates were incubated at 30°C and photographed after 72 h.

### Obtaining adaptors using a low amount of brefeldin A

Approximately 2.5 × 10^3^ cells/mL of H99 were inoculated into 1.5 mL of YPD broth containing 16 µg/mL BFA. After 72-h incubation with shaking, the culture was washed and diluted with distilled water, and approximately 200 cells were spread on YPD plates and incubated at 30°C for 72 h. A total of 120 colonies were randomly tested for tolerance to 64 μg/mL BFA.

### Obtaining adaptors using a high amount of brefeldin A

Cells were suspended in distilled water and adjusted to 1 × 10^7^ cells/mL. Cell suspension (100 µL) was spread on YPD plates supplemented with 64 μg/mL BFA. The plates were incubated at 30°C for 5 days. A total of 60 adaptors were randomly chosen. For each adaptor, four to six colonies of similar size were selected and frozen in 1 mL of 25% glycerol at −80°C.

### Genome instability assay

Aneuploid strains bearing different karyotypes [Chr1x2, Chr3x2, and Chrs(1,3)x2, respectively] were tested. The strains were streaked from a −80°C freezer to YPD agar and incubated at 30°C for 72 h. One small colony was randomly chosen and suspended in distilled water. Cells were diluted with distilled water and approximately 200 cells were spread on a YPD plate and incubated at 30°C for 72 h. One small (S) colony and one large (L) colony were randomly chosen for further studies.

### Next-generation sequencing

DNA extraction, library construction, and sequencing were performed as described previously (Yang et al., 2021c). Data were visualized using Ymap (Abbey et al., 2014). Raw fastq files were uploaded to Ymap (version 1.0) (http://lovelace.cs.umn.edu/Ymap/). Read depth was plotted as a function of chromosome position using the *C. neoformans* H99 reference genome (https://www.ncbi.nlm.nih.gov/assembly/GCF_000149245.1/).

### RNA-seq

Comparison between strains: RNA-seq was performed as described previously with slight modifications (Yang et al., 2021b). Briefly, strains were streaked on YPD plates from a −80°C freezer. After 72-h incubation at 30°C, several colonies with similar sizes were randomly chosen. Colonies were suspended at OD_600_ = 0.1. The cultures were incubated in a shaker at 30°C until OD_600_ reached 1.0. Cells were collected by centrifugation and were flash frozen in liquid nitrogen.

Treatment of H99 with BFA: H99 was grown in YPD broth in a shaker at 30°C from OD_600_ = 0.1 till 1.0. The culture was divided into two batches: in one batch, BFA at 32 μg/mL final concentration was added. In the other batch, the same volume of DMSO was added. Three hours later, the cells were collected by centrifugation and were flash frozen in liquid nitrogen.

Total RNA extraction and purification, library construction, and sequencing were performed as described previously (Sun et al., 2023a). Three biological replicates were obtained for each strain. Differential gene expression profiling was carried out using DESeq2 (Love et al., 2014) with standard parameters. Genes with FDR (false discovery rate)-adjusted *p*-value <0.05 and expression fold changes of more than 1.5 or less than −1.5 were considered differentially expressed.

### Strain construction

Gene deletions were performed as previously described (Hu et al., 2021). Overlapping PCR products were generated with the nourseothricin (NAT) resistance cassette and 5′ and 3′ flanking sequencings (1.0–1.5 Kb) of the target gene from wild-type H99. The deletion cassette was introduced into relevant *Cryptococcus* recipient strains by the TRACE method (Fan and Lin, 2018).

The overexpression vectors were constructed by assembling the following fragments: the open reading frames (ORFs) of genes were amplified by PCR; the plasmid pXC containing the P_CnH3_ promoter was digested by restrictive digestion enzymes FseI and PacI (Wang et al., 2012). The recombinant plasmids were generated using the ClonExpress Ultra One Step Cloning Kit (C115–02, Vazyme, China) and verified by sequencing. The overexpression sequences were amplified from the recombinant plasmids using SH2-F-PCR and SH2-R-PCR and introduced into the safe haven site (SH2) of *C. neoformans* by electroporation (Upadhya et al., 2017). Transformants were selected on geneticin plates and verified by PCR. Primers used in this study are listed in **Supplementary Table S1**.

### Real-time PCR

Cells were grown under the same experimental conditions as described in RNA-seq. RT-PCR was performed in 96-well plates (Bio-Rad) on the CFX Touch 96-well Real-Time Systems (Bio-Rad). Primer sequences are listed in **Supplementary Table S1**. The reaction mix was performed using 5 μL of iTaq Universal SYBR Green Supermix (Bio-Rad), 2 μL of 2 μM primer mix, 2 μL of a diluted 1:10 cDNA, and water to make up the final volume to 10 μL. Cycling conditions were 95°C for 3 min, 40 cycles of 95°C for 5 s, and 60°C for 30 min. Melt curve analysis conditions were 5 s at 95°C and then 5 s each at 0.5°C increments between 60°C and 95°C. *ACT1* was the internal control. The 2^−ΔΔCT^ algorithm was used to calculate the relative changes in gene expression (Livak and Schmittgen, 2001). All RT-PCR experiments were performed using three biological and three technical replicates.

### Statistical analysis

Significance analysis of differences between growth curves was performed using the Tukey HSD (Honestly Significant Difference) test.

## Results

### Short-time exposure to a low concentration of brefeldin A selects aneuploid resistant adaptors

**Figure 1:**
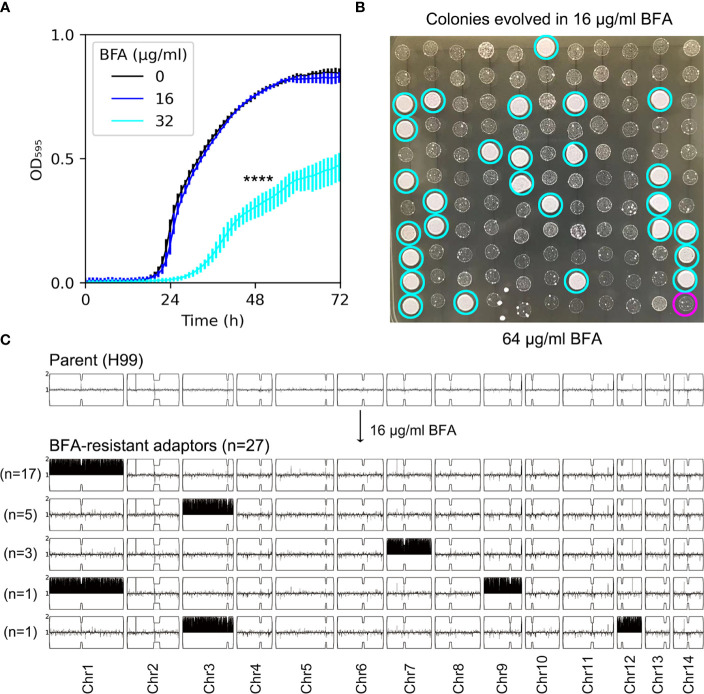
Short time exposure to a low amount of brefeldin A selects aneuploid adaptors. **(A)** Growth of H99 in YPD+BFA broth was monitored in a 96-well plate. The OD_595_ was measured at a time interval of 1 h for 72 h using a Tecan plate reader (Infinite F200 PRO, Tecan, Switzerland). Data are presented as the mean ± SD of three biological replicates. **** indicates *p*-values <0.0001 as determined by Tukey HSD test. **(B)** A total of 120 colonies pre-grown in YPD or YPD+BFA (16 μg/mL) were randomly tested for resistance by spot assay on a YPD+BFA plate. The magenta circle indicates the parent. Cyan circles indicate the 27 resistant colonies. The plates were incubated at 30˚C for 72 h and then photographed. **(C)** Both parent (top panel) and the 27 resistant colonies (bottom panel) were sequenced. The karyotypes were visualized using the Ymap. The chromosome copy number is indicated as a log_2_ ratio relative to that of the haploid H99 reference strain on the *y* axis, with one copy at the midline, clipped to show a maximum of two copies. The *x* axis shows the positions of the reads on each chromosome, mapped relative to the chromosome of reference strain H99.

The antifungal efficacy of BFA was evaluated by testing the growth of H99 in YPD broth supplemented with different concentrations of BFA. The growth curve of H99 in the absence of BFA was similar to the growth in the presence of 16 µg/mL BFA, indicating that this concentration was non-inhibitory. BFA (32 µg/mL) significantly inhibited the growth (*p*-value < 0.001, two-tailed paired *t*-test) (**Figure 1A**). We determined if short-time exposure to a sub-inhibitory amount of BFA would select resistant adaptors. After the growth in YPD broth supplemented with 16 μg/mL BFA, the culture was washed and diluted, then plated on a YPD plate without BFA. A total of 120 colonies were randomly compared to the parent for resistance to BFA by performing spot assay on a YPD plate with BFA. We found that 27 adaptors (**Figure 1B**, cyan circles) were more resistant than the parent (**Figure 1B**, magenta circle). As a control, none of the colonies pre-grown in YPD without BFA was resistant (data not shown). All the 27 BFA-resistant adaptors were sequenced. All adaptors were aneuploid: 17 adaptors had Chr1x2, 5 adaptors had Chr3x2, 3 adaptors had Chr7x2, 1 adaptor had Chr1x2+Chr9x2 [Chrs(1,9)x2], and 1 adaptor had Chr3x2+Chr12x2 [Chrs(3,12)x2] (**Figure 1C**). Therefore, BFA resistance can be rapidly induced by a sub-inhibitory concentration of BFA, and the resistance was mainly due to the formation of a particular aneuploidy including Chr1x2, Chr3x2, and Chr7x2.

### Supra-MIC concentration of brefeldin A selects aneuploid resistant adaptors

**Figure 2:**
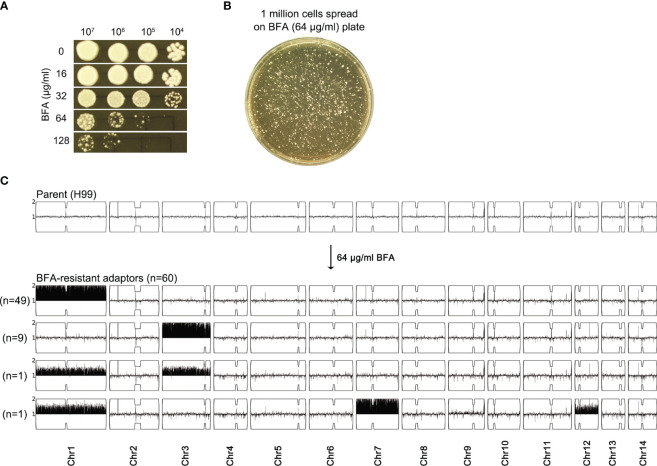
Exposure to a high amount of brefeldin A selects aneuploid adaptors. **(A)** Susceptibility of H99 to BFA on YPD-agar plates was evaluated by spot assay. Concentrations of BFA are shown in the figure. **(B)** Cells of H99 were spread on a YPD plate supplemented with 64 μg/mL BFA. A total of 60 adaptors were randomly chosen. **(C)** All adaptors were sequenced, and the karyotypes were visualized using Ymap. The number of adaptors bearing the same aneuploidy is indicated in the figure.

We determined how H99 adapted to a high concentration of BFA. On YPD-agar plates, the growth of H99 was obviously inhibited by 64 μg/mL BFA (**Figure 2A**). Approximately 1 million cells of H99 were spread on YPD plates supplemented with 64 μg/mL BFA (**Figure 2B**). A total of 60 adaptors were randomly chosen. Spot assay indicated that all adaptors were more resistant to BFA than the parent (**Supplementary Figure S1**). Whole-genome sequencing indicated that all adaptors were aneuploid: 49 adaptors had Chr1x2, 9 adaptors had Chr3x2, 1 adaptor had Chr1x2 and Chr3x2 [Chrs(1,3)x2], and 1 adaptor had Chr1x2+Chr7x2+Chr12x2 [Chrs(1,7,12)x2] (**Figure 2C**). Therefore, BFA resistance induced by a high concentration of BFA was also mainly due to the formation of Chr1x2, Chr3x2, and Chr7x2.

### Aneuploidy confers cross-heteroresistance to brefeldin A and antifungal drugs

**Figure 3:**
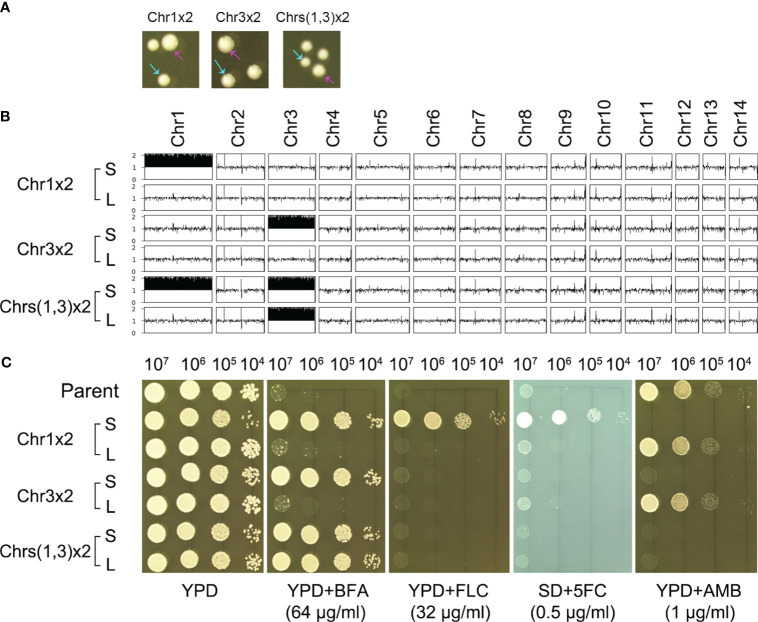
Aneuploidy confers heteroresistance. **(A)** Three aneuploids were spread on a YPD plate. The karyotypes are indicated in the figure. Colony instability was evidenced by the appearance of small (indicated by cyan arrows) and large colonies (indicated by magenta arrows). **(B)** The large colonies were sequenced, and the karyotypes were visualized using Ymap. **(C)** For each aneuploid, one small colony (S) and one large colony (L) were tested for resistance to BFA and antifungal drugs fluconazole (FLC), 5-flucytosine (5FC), and amphotericin B (AMB). Spot assay was performed. Three microliters of 10-fold serial dilutions of each test strain was spotted on YPD plates with or without drugs. SD plates were used when 5FC was tested. The plates were incubated at 30°C for 72 h and then photographed.

We determined if the resistance was stable. One adaptor with Chr1x2 (TJ1952), one adaptor with Chr3x2 (TJ1969), and one adaptor with Chrs(1,3)x2 (TJ1985) were randomly tested for genomic and phenotypic stability. When grown on YPD plates, all three adaptors exhibited colony size instability, indicated by small-sized colonies (**Figure 3A**, cyan arrows) and large-sized colonies (**Figure 3A**, magenta arrows). For each adaptor, one small colony [Chr1x2-S, Chr3x2-S, and Chrs(1,3)x2-S] and one large colony [Chr1x2-L, Chr3x2-L, and Chrs(1,3)x2-L] were further randomly tested. Whole-genome sequencing indicated that Chr1x2-S, Chr3x2-S, and Chrs(1,3)x2-S had the same karyotype as the progenitors. Chr1x2-L and Chr3x2-L were euploids, and Chrs(1,3)x2-L had Chr3x2 (**Figure 3B**). We determined if Chrs(1,3)x2 would always become Chr3x2 instead of Chr1x2 in the absence of stress. An additional nine large colonies were randomly sequenced, and all were Chr3x2 (data not shown). Grown on a YPD plate supplemented with BFA, all small colonies were resistant to BFA. Chr1x2-L and Chr3x2-L were not resistant, but Chrs(1,3)x2-L was still resistant (**Figure 3C**). Therefore, the genomes of the aneuploid adaptors were unstable in the absence of stress. Aneuploids spontaneously and rapidly reverted to euploids. In the adaptor with aneuploidy of two chromosomes, the larger extra chromosome tended to be lost first. Resistance to BFA in the aneuploids was due to the altered chromosome copy number.

We also determined if exposure to BFA altered the antifungal resistance profile. Grown on a YPD plate supplemented with antifungal drugs, compared to the parent, Chr1x2-S was more resistant to FLC and 5FC, but more susceptible to AMB. Chr3x2-S was also more susceptible to AMB. Chr1x2-L and Chr3x2-L had a similar drug resistance profile to the parent (**Figure 3C**). In addition to spot assay, disk diffusion assays were also performed. On agar plates with disks containing FLC or 5FC, the Chr1x2 strain had an obviously smaller inhibition zone than the parent, while the Chr3x2 strain had a similar size of inhibition zone to the parent (**Supplementary Figure S2**). In addition, all the 60 supra-MIC BFA-induced adaptors were tested for resistance to different antifungal drugs. All adaptors were hypersensitive to AMB, and all Chr1x2 adaptors were resistant to FLC and 5FC (**Supplementary Figure S3**). Thus, Chr1x2 enabled multidrug heteroresistance to BFA, FLC, and 5FC; Chr3x2 conferred heteroresistance only to BFA; and both Chr1x2 and Chr3x2 were hypersensitive to AMB.

### Particular genes on Chr1 and Chr3 are associated with drug resistance

**Figure 4:**
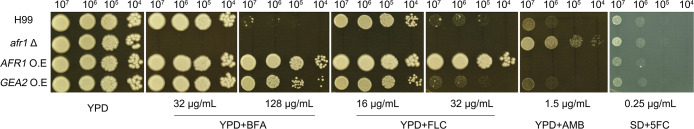
Role of *AFR1* and *GEA2* in drug resistance. The wild type was H99. *AFR1* deletion, *AFR1* overexpression, and *GEA2* overexpression strains were constructed. The constructs were compared to H99 for resistance to BFA, FLC, AMB, and 5FC. SD medium was used for testing 5FC, and YPD was used for other tests. The plates were incubated at 30˚C for 72 h and then photographed.

To evaluate the role of *AFR1* and *GEA2* in resistance to different drugs, strains with deletion and overexpression of *AFR1*, as well as overexpression of *GEA2*, were constructed. Spot assays were performed. The wild-type H99 could tolerate 32 μg/mL BFA. *AFR1* deletion strain was obviously inhibited at this concentration. *AFR1* overexpression strain and *GEA2* overexpression strain could tolerate 128 μg/mL BFA (**Figure 4**). Both H99 and *GEA2* overexpression strain could tolerate 16 μg/mL FLC, and both were inhibited by 32 μg/mL FLC. *AFR1* deletion strain was obviously inhibited by 16 μg/mL FLC, while *AFR1* overexpression strain could tolerate 32 μg/mL FLC (**Figure 4**). Thus, both *AFR1* and *GEA2* were associated with BFA resistance. *AFR1* was also associated with FLC resistance. Interestingly, deletion of *AFR1* conferred increased resistance to AMB, and overexpression of *AFR1* caused hypersensitivity to AMB (**Figure 4**). Finally, deletion or overexpression of *AFR1* and overexpression of *GEA2* did not cause obvious change of resistance to 5FC (**Figure 4**).

### *AFR1* is required for brefeldin A-induced formation of chromosome 1 disomy

**Figure 5:**
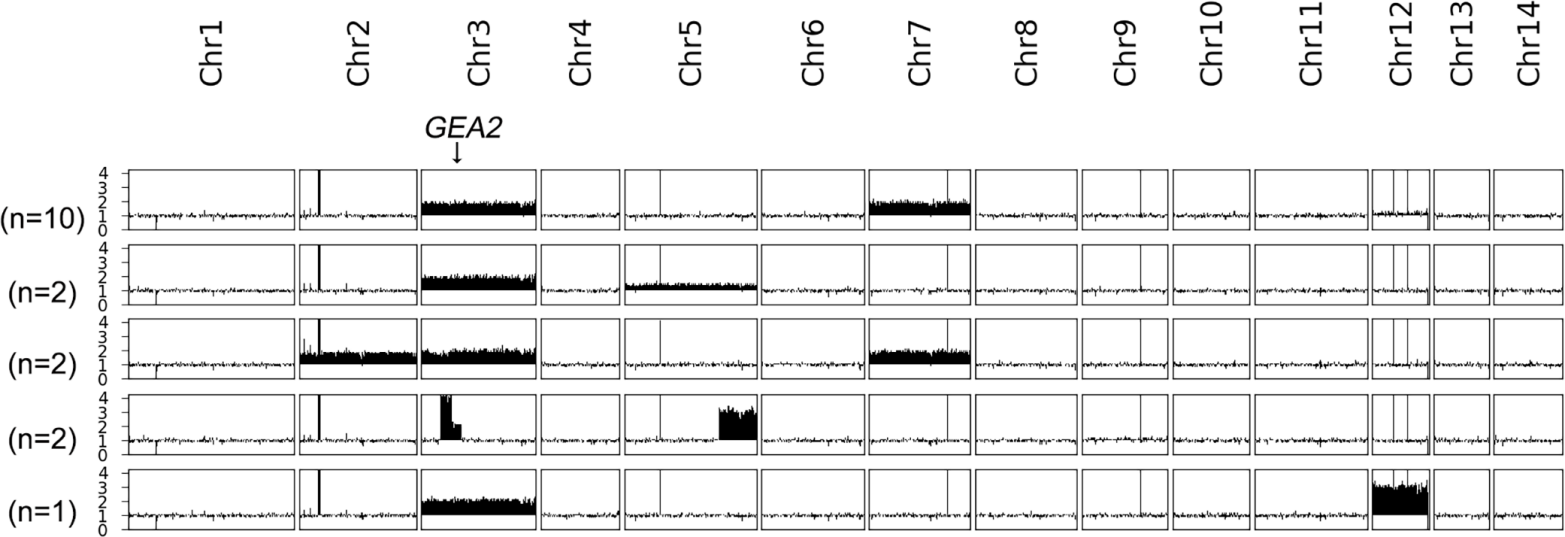
Karyotypes of *AFR1* deletion strain-derived brefeldin A adaptors. *AFR1* deletion strain was used as a parent to obtain BFA adaptors. A total of 17 resistant adaptors were randomly sequenced. The locus of *GEA2* on Chr3 is indicated by an arrow in the figure. On the left of the karyotypes, the number of adaptors bearing the karyotype was shown.

*AFR1* deletion strain was spread on a YPD plate supplemented with 8 μg/mL BFA. A total of 17 resistant adaptors were randomly sequenced (**Figure 5**). All of them were aneuploid: 10 adaptors had Chrs (3,7)x2, 2 adaptors had Chrs (3,5)x2, 2 adaptors had Chrs (2,3,7)x2, and 1 adaptor had Chr3x2+Chr12x3. Interestingly, instead of whole chromosome aneuploidy, two adaptors had segmental amplification on Chr3 and Chr5. The amplification on Chr3 resulted in tetrasomy of the region from 0.269 Mb to 0.423 Mb, and disomy from 0.423 Mb to 0.551 Mb. *GEA2* was in the disomic region. The amplification on Chr5 caused trisomy of the region from 1.309 Mb to the right telomere. Thus, *AFR1* deletion strain adapted to BFA via segmental or whole chromosome duplication of Chr3, which contains *GEA2* in combination with disomy or trisomy of other chromosomes.

### Pleotropic effect of aneuploidy on phenotypes is due to elevated expressions of related genes

**Figure 6:**
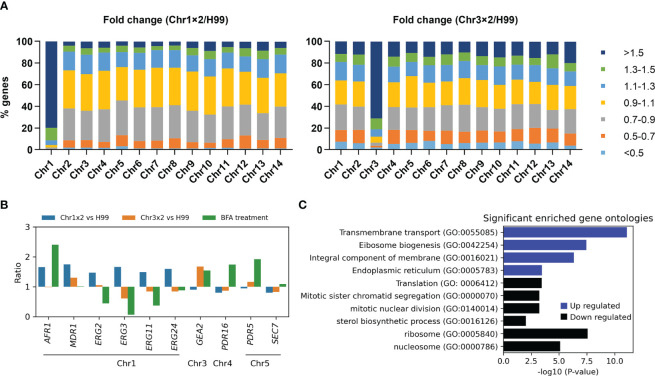
Transcriptional consequences of aneuploidy. **(A)** Transcriptomes of Chr1x2 and Chr3x2 strains were compared to H99. Genes were categorized into different groups based on the ratios. Numbers of genes belonging to each group were presented as percentage of the total number of genes on the chromosome. **(B)** Comparative transcriptome analysis of some genes was presented. Chr1x2/H99 and Chr3x2/H99 indicate ratios of transcripts of genes in Chr1x2 and Chr3x2 strains as compared to H99, respectively. “BFA treatment” indicates the ratio of transcripts of the genes in BFA-treated cells as compared to no treatment. **(C)** Gene ontology (GO) enrichment analysis of differential genes in H99 treated with BFA as compared to no treatment. The analysis was performed using FungiDB (https://fungidb.org/fungidb/app). Up- and downregulated genes were analyzed separately.

To investigate why particular aneuploidy conferred cross-resistance to antifungals, transcriptomes of one Chr1x2 adaptor (TJ1952) and one Chr3x2 adaptor (TJ1669) were compared to the parent. In the Chr1x2 adaptor, among the 858 genes on Chr1, 687 (80.0%), 747 (87.1%), 785 (91.5%), and 808 (94.2%) genes had ratios of relative expression to wild type higher than 1.5, 1.4, 1.3, and 1.2, respectively. In the Chr3x2 adaptor, among the 589 genes on Chr3, 421 (71.5%), 450 (76.4%), 479 (81.3%), and 498 (84.5%) genes had ratios higher than 1.5, 1.4, 1.3, and 1.2, respectively. In total, there were 961 and 1,094 significantly upregulated genes in Chr1x2 (TJ1952) and Chr3x2 (TJ1969) adaptors, respectively. Of the upregulated genes in Chr1x2 adaptor, 70.7% (679 out of 961) were on Chr1, and 37.5% (410 out of 1,094) of the upregulated genes in Chr3x2 adaptor were on Chr3. There were 370 and 486 significantly downregulated genes in Chr1x2 and Chr3x2 adaptors, respectively. Only 0.5% (2 out of 370) of downregulated genes in Chr1x2 adaptor were on Chr1, and 4.1% (20 out of 486) of downregulated genes in Chr3x2 adaptor were on Chr3 (**Figure 6A**). Thus, in general, aneuploidy directly caused elevated expression of most genes on the aneuploid chromosome and indirectly regulated expression of a few genes on euploid chromosomes.

To analyze cellular response to BFA treatment in the wild-type strain, RNA-seq of H99 cells treated with an inhibitory concentration of BFA (test) or an equal amount of vehicle (control) was performed. Here, we found exposure to BFA induced expression of *GEA2* but not *SEC7* (**Figure 6B**). We also found, in Chr3x2 adaptor, but not in Chr1x2 adaptor, that expression of *GEA2* was also higher than that in H99 (**Figure 6B**). In addition, RT-PCR indicated that the relative expressions of *GEA2* in Chr3x2 and the *GEA2* overexpression strain were 2.79 ± 0.25 and 9.27 ± 0.25, respectively, as compared to H99. Thus, we posit that Chr3x2 causes BFA resistance via increasing copy number, thereby increasing expression of *GEA2*.

BFA is substrate of efflux pumps (Diwischek et al., 2009). In *C. neoformans* genome, several genes encode efflux pumps, including Chr1 genes *AFR1/CNAG_00730* and *MDR1/CNAG_00796*, Chr5 gene *PDR5/CNAG_00869*, and Chr4 gene *PDR16/CNAG_04984*. Here, we found exposure to BFA induced expression of *AFR1*, *PDR5*, and *PDR16*, but not *MDR1*. Compared to H99, in Chr1x2 adaptor, expressions of *AFR1* and *MDR1*, but not *PDR5* or *PDR16*, were higher, but in Chr3x2 adaptor, none of them were differentially expressed (**Figure 6B**). RT-PCR indicated that the relative expressions of *AFR11* in Chrx2 and the *AFR1* overexpression strain were 1.81 ± 0.11 and 3.58 ± 0.33, respectively, as compared to H99. Thus, we posit that Chr1x2 causes BFA resistance via increasing copy number, thereby increasing expression of *AFR1*. However, since the relative expressions of both *AFR1* and *GEA2* in aneuploids were lower than in the overexpression strains, we cannot exclude the possibility that, in addition to *AFR1* and *GEA2*, aneuploidy might upregulate other genes on the aneuploid chromosomes (direct regulation) or on euploid chromosomes (indirect regulation), which also contribute to BFA resistance. A previous study by Hu et al. found that deletion of *CDC50/CNAG_06465* conferred hypersensitivity to BFA (Hu et al., 2017). *CDC50* is on Chr13. In this study, aneuploidy of Chr13 was not detected. Furthermore, in both Chr1x2 and Chr3x2 adaptors, the expression of CDC50 was comparable to H99. Thus, aneuploidy-mediated regulation of CDC50 copy number or expression was not a major mechanism of adaptation to BFA in this study.

It is known that exposure of H99 to FLC selects Chr1x2 adaptors, and Chr1x2 causes resistance to FLC via upregulating *AFR1* and *ERG11* genes on Chr1 (Sionov et al., 2010). On Chr1, there are other genes probably associated with FLC resistance, including *MDR1*, *ERG2/CNAG_00854*, *ERG3/CNAG_00519*, and *ERG24/CNAG_00117*. Here, we found, among these genes, that exposure to BFA caused only higher expression of *AFR1* and caused downregulation of *ERG2*, *ERG3*, and *ERG11*, while in Chr1x2 adaptor, as compared to H99, all of these genes were upregulated (**Figure 6B**). Thus, we posit that BFA-induced Chr1x2 confers resistance to FLC via upregulating *AFR1*, *ERG11*, and other efflux genes (e.g., *MDR1*) and *ERG* genes (e.g., *ERG2*, *ERG3*, and *ERG24*).

RNA-seq indicates that BFA induces both UPR and a defective cell cycle. Induction of chaperon genes and attenuation of global translation are reminiscent of UPR (Naidoo, 2009). Aneuploidy formation usually results from chromosome mis-segregation. Repression of mitotic genes leads to increased chromosome mis-segregation and aneuploidy formation (Macedo et al., 2018). Here, we found, upon exposure of H99 to BFA, that several genes encoding ER chaperones were significantly upregulated (ratio > 1.5, *q* < 0.05), including *ERO1/CNAG_03176*, *SCJ1/CNAG_05252*, *FMO1/CNAG_00541*, *CNE1/CNAG_02500*, and *FES1/CNAG_01185*. Several other genes encoding chaperones were also upregulated, including *HSP26/CNAG_05706*, which encodes small heat shock proteins that suppress unfolded protein aggregation, and *KAR2/CNAG_06443*, *DFM1/CNAG_06365*, *GET3/CNAG_01923*, *SDH8/CNAG_02149*, and *BCP1/CNAG_03897*. GO enrichment analysis indicated that localization of products of the 927 upregulated genes was significantly enriched in ER, while products of the 1,005 downregulated genes were significantly enriched in the ribosome and nucleosome. The upregulated genes were significantly enriched in biological processes including transmembrane transport and ribosome biogenesis. The downregulated genes were significantly enriched in biological processes including translation, mitotic sister chromatid segregation, mitotic nuclear division, and sterol biosynthetic process (**Figure 6C**).

## Discussion

Stresses are a normal part of life for all living organisms. *C. neoformans* is no exception. In the environment, *C. neoformans* faces diverse environmental stresses, such as osmotic shock, oxidative stress, and genotoxic stress. In the human host, it suffers from oxidative and nitrosative stress, high temperature, hypoxia, and nutrient deprivation. Investigations on mechanisms of antifungal resistance have been limited to studies of the interactions between organisms and drugs. However, the impact of non-antifungal drugs on the development of antifungal resistance in *C. neoformans* is largely unexplored. Historically, ER stress caused by chemical inductions is the focus of investigations, which are limited to dissecting the signaling pathways. This study demonstrates for the first time that BFA-induced ER stress has the potential of causing resistance to antifungal drugs via aneuploidy in *C. neoformans*. Our study also sheds new light on the origin of antifungal drug resistance: being exposed to the drug does not have to be the prerequisite. Adaptation to one stress (e.g., ER stress) might cause cross-adaptation to other unrelated stresses (e.g., antifungal drugs). This principle is also applicable to *C. albicans* (Yang et al., 2019; Yang et al., 2021b) and *C. parapsilosis* (Yang et al., 2021d; Sun et al., 2023a).

Adaptation to stresses can be due to altered gene sequence and/or altered gene copy number. The latter can be due to gene amplification, segmental, whole chromosome aneuploidy, or even ploidy shift (Bennett et al., 2014). Aneuploidy has the potential of simultaneously regulating copy number and expression of hundreds of genes on the aneuploid chromosome, as well as genes on euploid chromosomes (Pavelka et al., 2010). Thus, aneuploidy is considered a rapid strategy for evolution of pleiotropic traits. Here, we found that both weak and strong selection by BFA mainly selected Chr1x2 or Chr3x2. In *S. cerevisiae*, overexpression of *GEA2* and *SEC7* genes, which encode BFA targets, confers resistance to BFA (Peyroche et al., 1999). The ortholog of *S. cerevisiae GEA2* in *C. neoformans* is *GEA2/CNAG_07514*, which is on Chr3. The ortholog of *S. cerevisiae SEC7* in *C. neoformans* is *SEC7/CNAG_01126*, which is on Chr5. BFA is a substrate of efflux pump (Diwischek et al., 2009), and *AFR1* on Chr1 is a well-known efflux pump encoding gene (Sionov et al., 2010). Transcriptome analysis also indicated that exposure of H99 to BFA induced expression of *GEA2* and *AFR1*, along with other genes involved in ER stress. Our study also indicated that overexpression of *AFR1* conferred resistance to BFA, and deletion of *AFR1* caused hypersensitivity. Furthermore, *AFR1* deletion strain was unable to form Chr1x2 in response to BFA. Thus, we posit that *AFR1* is the driving force of selection of Chr1x2 upon BFA treatment. Overexpression of *GEA2* conferred BFA resistance. We were unable to construct GEA2 deletion strain since it is an essential gene, and we could not test if *GEA2* is the determinant gene of Chr3x2 formation upon BFA treatment. Transcriptome analysis also indicated that Chr1x2 simultaneously upregulated expression of *AFR1*, which is associated with resistance to 5FC and FLC, as well as other genes associated with resistance to FLC, such as *MDR1* and several *ERG* genes. Chr1x2 and Chr3x2 also altered expression of multiple genes on euploid chromosomes. We posit that the pleiotropic new traits caused by Chr1x2, e.g., cross-resistance to BFA, 5FC, and FLC, are due to the direct and indirect effect of aneuploidy on expression of genes on aneuploid and euploid chromosomes.

It is well known that aneuploidy is a prevalent strategy for the rapid adaptation to antifungal drugs in pathogenic fungi including *C. neoformans* and *Candida* spp (Selmecki et al., 2006; Sionov et al., 2010; Yang et al., 2017; Yang et al., 2019; Bing et al., 2020; Yang et al., 2021a; Burrack et al., 2022; Sun et al., 2023a; Sun et al., 2023b). ER is the site for the synthesis of sterols and lipids, and in lower eukaryotes including *Cryptococcus* spp. and *Candida* spp., the site for synthesis of the major proportion of the cell wall [reviewed in (Schroder and Kaufman, 2005)]. We posit that antifungal drugs that target the cell membrane (i.e., polyenes and azoles) or the cell wall (i.e., echinocandins) might cause aneuploidy directly or indirectly via induction of ER stress.

The finding that both Chr1x2 and Chr3x2 caused hypersensitivity to AMB is very intriguing. The emergence of *Candida* spp. with AMB resistance is rare, despite >50 years of clinical use. Experimental evolution of AMB resistance is accompanied with fitness trade-offs (Vincent et al., 2013). Thus, a clear mechanism of AMB resistance is still lacking. A generally accepted mechanism is that AMB resistance can be acquired through mutations in ergosterol biosynthesis genes, which result in the depletion of ergosterol and accumulation of alternate sterols. Accordingly, mutations of some *ERG* genes have been linked to AMB resistance in *Candida* spp., such as *ERG2* and *ERG6* in *C. glabrata* (Ahmad et al., 2019), and *ERG3* in *C. albicans* (Kelly et al., 1997; Nolte et al., 1997). However, here, we found that defective efflux was associated with AMB resistance in *C. neoformans*: deletion of *AFR1* caused resistance, and overexpression caused hypersensitivity to AMB. In addition, we found that deletion of the major efflux gene *CDR1* in *C. albicans* also conferred AMB resistance (Feng Yang, unpublished data). Membrane lipid constituents affect positioning and functional maintenance of the integral efflux proteins in *C. albicans* (Pasrija et al., 2005), but the effect of deletion of efflux gene on membrane lipid composition is largely unexplored. We posit that deletion and overexpression of *AFR1* might result in re-organization of lipid constituents in the membrane, thereby altering the ability of AMB binding to membrane lipids.

Importantly, to induce aneuploidy formation, the selective force does not have to be strong, and the exposure duration does not have to be long. In this study, we found that 72-h exposure to sub-inhibitory amount of BFA was sufficient to select aneuploids in *C. neoformans*. Previously, we also found that 24-h exposure to weak ER stress induced by TUN was sufficient to induce aneuploidy in *C. albicans* (Yang et al., 2021b). Short time exposure to a sub-inhibitory concentration of FLC, e.g., 72 h and 24 h for *C. neoformans* and *C. albicans*, respectively, also selected aneuploids (Yang et al., 2021a; Sun et al., 2023b).

By convention, genome instability refers to situations in which euploid organisms lose fidelity of chromosome segregation. Here we want to emphasize genome instability from a different angle: in the absence of stress, aneuploids usually fail to maintain the aneuploid state and spontaneously become euploids. In fact, the instability of aneuploids has been well documented in *C. neoformans* (Sionov et al., 2010) and in *C. albicans* (Yang et al., 2013; Yang et al., 2021c), and is also found in other yeasts (Bing et al., 2020). We posit that genome instability might be the cause of rare detection of aneuploid clinical isolates of *C. neoformans*. However, aneuploidy has been detected in freshly isolated isolates (Hu et al., 2011; Stone et al., 2019).

In summary, we found that BFA, the commonly used chemical inducer of ER stress, mainly selected particular and transient aneuploidy (Chr1x2 or Chr3x2) in *C. neoformans*. Chr1x2 simultaneously upregulated multiple genes associated with resistance to unrelated drugs, thereby causing cross-resistance. In mammalian cells, many physiological conditions induce ER stress [reviewed in (Schroder and Kaufman, 2005)]. Although whether aneuploidy is the driver or consequence of tumorigenesis is still under debate (Ben-David and Amon, 2020), it will be interesting to test if weak or strong ER stress induces aneuploidy formation in mammalian cells.

## Data availability statement

The datasets presented in this study can be found in online repositories. The names of the repository/repositories and accession number(s) can be found in the article/**Supplementary Material**.

## Author contributions

FY: Writing – review & editing, Visualization, Formal analysis. Z-hZ: Writing – review & editing, Visualization, Validation, Methodology, Investigation, Formal analysis, Data curation. L-lS: Writing – review & editing, Visualization, Validation, Investigation. B-qF: Writing – review & editing, Visualization, Investigation, Data curation. JD: Writing – review & editing, Investigation, Data curation. C-lJ: Writing – review & editing, Investigation, Formal analysis. M-xM: Writing – review & editing, Formal analysis, Data curation. Y-bC: Writing – review & editing, Supervision, Resources, Project administration, Funding acquisition, Conceptualization. T-hY: Writing – review & editing, Writing – original draft, Supervision, Resources, Project administration, Conceptualization.
