# Supplementary material for: Aneuploidy underlies brefeldin A-induced antifungal drug resistance in Cryptococcus neoformans
Source: Front Cell Infect Microbiol. 2024 Jun 20;14:1397724. doi: 10.3389/fcimb.2024.1397724 (PMC11222406; doi:10.3389/fcimb.2024.1397724)
Supplement: Supplementary file 1 [file DataSheet_1.docx]

**Supplementary materials**

**Figure S1. Supra-MIC brefeldin A adaptors are resistant to BFA**

All 60 randomly selected adaptors (TJ1952-TJ2011) were tested for resistance to BFA by spot assay. 3 µL of 10-fold serial dilutions were spotted on the plates. The plates were incubated at 30˚C and photographed after 72 h.

**Figure S2. Evaluation of resistance to fluconazole and 5-flucytosine by disk diffusion assay**

The wild type strain H99, one Chr1x2 adaptor (TJ1952) and one Chr3x2 adaptor (TJ1669) were tested with disk diffusion assays. YPD plates were used for testing fluconazole (FLC), and SD plates were used for testing 5-flucytosine (5FC). The amounts of FLC and 5FC in the disks were 200 μg and 0.5 μg, respectively. The plates were incubated at 30˚C for 72h then photographed.

**Figure S3. Testing supra-MIC brefeldin A adaptors for resistance to antifungal drugs**

All the 60 adaptors as described in Fig S1 were tested for resistance to fluconazole (FLC), amphotericin B (AMB) and 5-flucytosine (5FC). YPD medium was used for testing FLC and AMB. SD medium was used for testing 5FC. The plates were incubated at 30˚C for 72h then photographed.

**Table S1. Primers used in this study**

**Table S2. GO enrichment of differential genes in H99 treated with BFA.**
