# Supplementary figures and images for: Aneuploidy underlies brefeldin A-induced antifungal drug resistance in Cryptococcus neoformans
Source: Front Cell Infect Microbiol. 2024 Jun 20;14:1397724. doi: 10.3389/fcimb.2024.1397724 (PMC11222406; doi:10.3389/fcimb.2024.1397724)

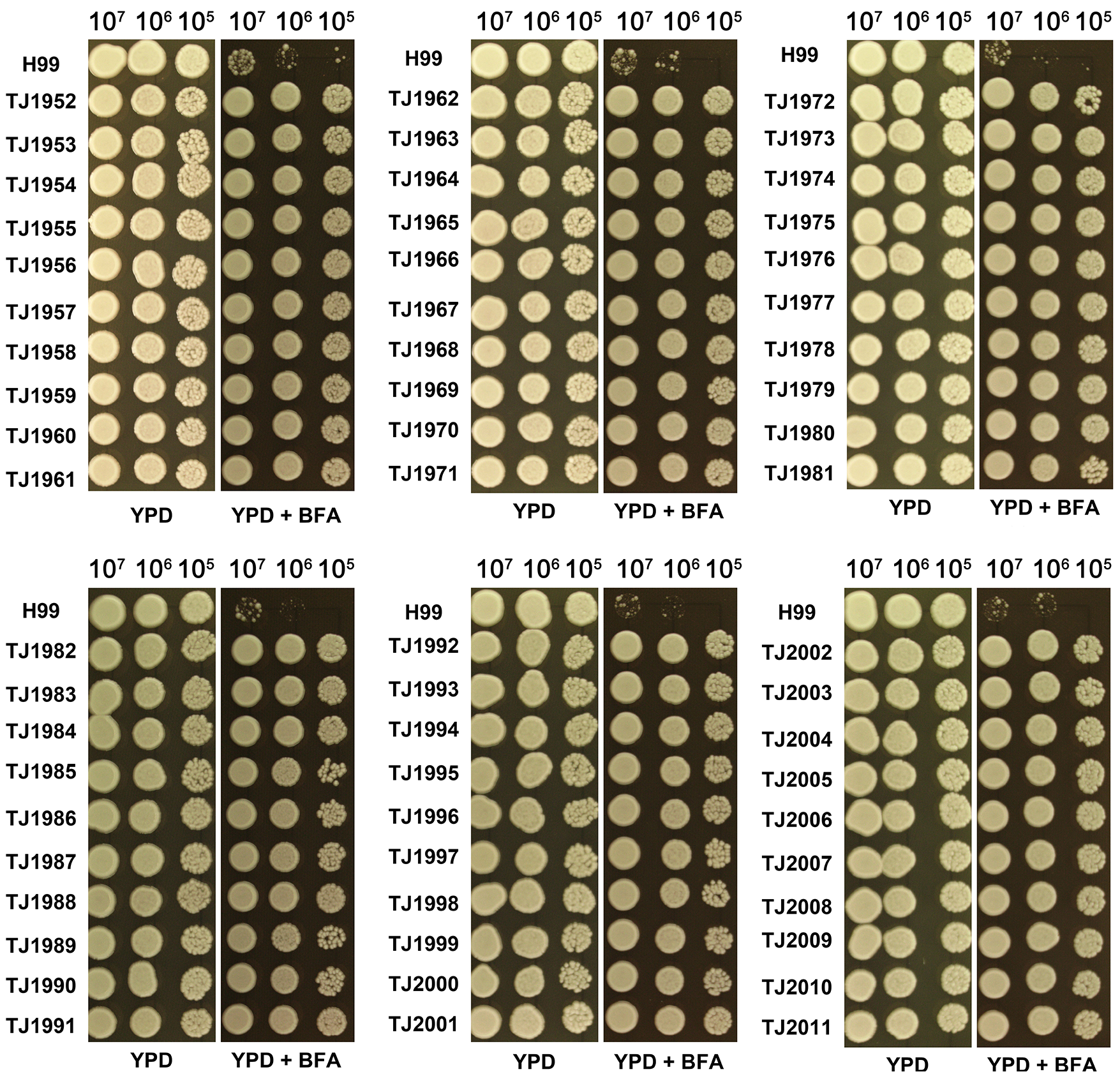

Supplement: Supplementary file 2 [file Image_1.tif]

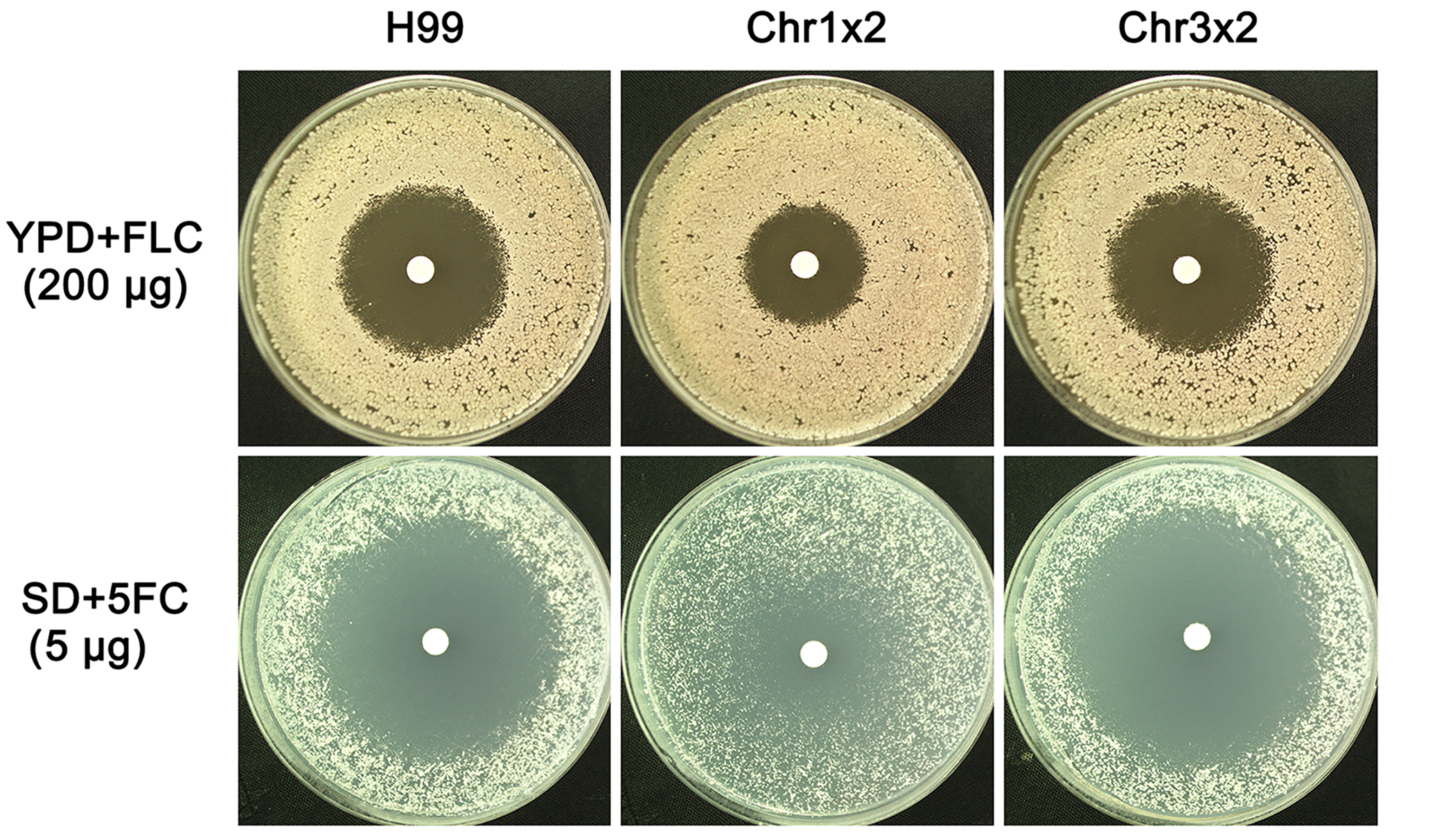

Supplement: Supplementary file 3 [file Image_2.tif]

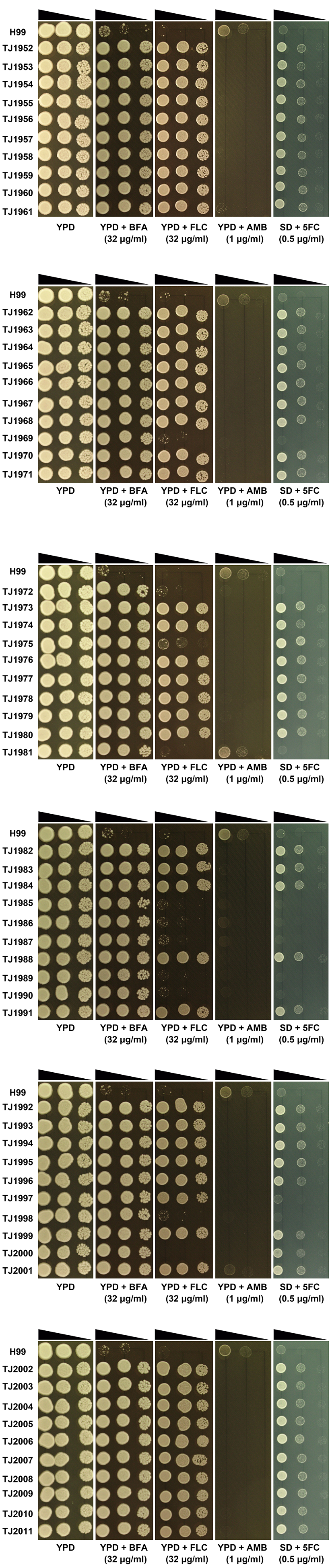

Supplement: Supplementary file 4 [file Image_3.tif]
