# Supplementary material for: Aneuploidy underlies brefeldin A-induced antifungal drug resistance in Cryptococcus neoformans
Source: Front Cell Infect Microbiol. 2024 Jun 20;14:1397724. doi: 10.3389/fcimb.2024.1397724 (PMC11222406; doi:10.3389/fcimb.2024.1397724)
Supplement: Supplementary file 5 [file Table_1.docx]

Table S1. Primers used in this study

| Name | Sequence (5′-3′) |
| --- | --- |
| Primers for overexpressions | |
| AFR1-F (Fse1) | agtacatttatcacaggccggccATGTCAGCTGCAGGCGTTCC |
| AFR1-R (Pac1) | ctgctactgtaacccttaattaaTTATCTCCTCTGCCACTTCAAAAA |
| AFR1-R (Asis1) | cccttgctcaccattgcgatcgcTCTCCTCTGCCACTTCAAAAA |
| GEA2-F (Fse1) | agtacatttatcacaggccggccATGATGCCGCCTACAGATCTCC |
| GEA2 (R (Pac1) | ctgctactgtaacccttaattaaCTAAGACTGCTCTACTTGCTGCTCA |
| GEA2-R (Asis1) | cccttgctcaccattgcgatcgcAGACTGCTCTACTTGCTGCTCAAC |
| SH2-F-PCR | ACAAAGCCGCAAGGATGG |
| SH2-R-PCR | CCGCCCTCCTTTACTACC |
| SH2-Test-F | CTGTAGAAGAGCGAATAACCTT |
| SH2-Test-R | AGCTGTGACAACTTGGC |
| Primers for deletions | |
| U6 promoter F | CCATCGATTTGCATTAGAACTAAAAACAAAGCA |
| gRNA R | CCGCTCGAGTAAAACAAAAAAGCACCGAC |
| CnAFR1-US-F | ACTGCCCTTCATTGTGGACCTCT |
| CnAFR1-US-R | CTGGCCGTCGTTTTACACTTACTGCTATTGCCGCTTGCT |
| CnAFR1-DS-F | GTCATAGCTGTTTCCTGTTTGCCGTTAGGAGGGTGGAGTT |
| CnAFR1-DS-R | TGTCTACATCGTGCTTTGTGCTG |
| CnAFR1_sgRNA-U6-R | CTGGTCTCGATGGACAGAGCAACAGTATACCCTGCCGGTG |
| CnAFR1_sgRNA-F | GCTCTGTCCATCGAGACCAGGTTTTAGAGCTAGAAATAGCAAGTT |
| CnAFR1-Split-F | caagggctggacacttatgagat |
| CnAFR1-Split-R | tagccaaagaaaggacagaagga |
| DRUG MARKER SE 1 | GTAAAACGACGGCCAGTGC |
| DRUG MARKER AN 1 | CAGGAAACAGCTATGACATGATTAC |
| G418-F | GGATCAAGCGTATGCAGC |
| G418-R | TCCATCTCGCCTTGTCC |
| Primers for RT-PCR | |
| ACT1-F | GGTATGTGCAAGGCTGGTTT |
| ACT1-R | CTTCTCCATGTCGTCCCAGT |
| AFR1-F | GGTTCCGACTACATGGCTGT |
| AFR1-R | GAGTTCACCAGCTCGGAAAG |
| GEA2-F | TCCAATGAAGACTGCTGTGC |
| GEA2-R | ACAATCTACCGCCACCAAAG |
